# Nitrogen fertilization application strategies improve yield of the rice cultivars with different yield types by regulating phytohormones

**DOI:** 10.1038/s41598-023-48491-w

**Published:** 2023-12-09

**Authors:** Yue Zou, Yuchen Zhang, Jiehao Cui, Jiacong Gao, Liying Guo, Qiang Zhang

**Affiliations:** 1 Agronomy College Jilin Agricultural University, Changchun, 130118 China; 2 Key Laboratory of Germplasm Innovation and Physiological Ecology of Grain Crops in Cold Region, Ministry of Education, Harbin, 150030 China

**Keywords:** Plant hormones, Plant physiology

## Abstract

Rice (*Oryza sativa L.*) is the most important food crop worldwide, and its sustainable development is essential to ensure global food security. Panicle morphological and physiological characteristics plays an important role in rice yield formation. However, under different nitrogen (N) fertilization strategies, it is not clear whether the morphological and physiological state of panicles at panicle development stage affects the formation of yield. To understand how the panicle differentiation and development, and grain yield are affected by the N fertilization strategies, and clarify the relationship between related traits and yield in the process of panicle development in different cultivars. In this study consisted of no N fertilizer and four N fertilization strategies, a panicle weight type (PWT) rice cultivar, Dongfu 114 (DF114) and a panicle number type (PNT) rice cultivar, Longdao 11 (LD11) were grown in the field. The results showed that N fertilization strategies could improve the nitrogen use efficiency and yield of rice, but the response of different rice varieties to N fertilizer strategies was different. Different from the DF114, the further increase of panicle N fertilizer ratio could not further improve the yield of LD11, and the highest grain yield of DF114 and LD11 was obtained under N4 and N3 conditions, respectively. In addition, this study found that N fertilizer strategies can affect the content of phytohormones in rice at the panicle differentiation stage, and then regulate the differentiation and development of rice panicles to affect yield. It is of great significance to optimize the application mode of N fertilizer according to the characteristics of varieties to improve rice yield and ensure food security.

## Introduction

Rice (*Oryza sativa L.*) is one of the most important food crops in the world, about half of the world's people use rice as their staple food. Its sustainable and healthy development is an important support to ensure world's food security. Under the severe situation of increasing population and decreasing land, people have steadily improved the yield of rice by improving rice cultivars, advancing cultivation techniques and increasing production inputs, among them, increasing nitrogen (N) input is one of the effective measures^1–3^. N is one of the most important nutrient elements, which can greatly affect the yield of rice. Since the last century, due to the increase of N application, rice yield has also increased significantly. However, rice plants can only use part of the applied N fertilizer, accounting for about 20–30% of the total amount of fertilizer, excessive and unreasonable N fertilizer input has also brought a series of problems such as unstable grain production, decreased nitrogen use efficiency (NUE), increased production costs, exacerbating environmental pollution, which has seriously affected the sustainable development of rice production^4–8^.

In view of the above problems, many scholars have carried out a lot of research on how to improve the NUE in paddy fields, and have made great progress in the comparison, screening, evaluation and utilization of NUE of rice germplasm, the physiological and biochemical characteristics, root morphology, dry matter production and accumulation characteristics of different nitrogen use efficiency genotypes, and the effects of field N management on rice yield, quality and NUE^9–15^. In addition, under the context of sustainable agricultural production, appropriate N management measures are needed to protect the environment and increase rice productivity. Over the past few decades, many optimized nitrogen fertilization strategies have been proposed and tested in field trials, such as top dressing, precise quantitative fertilization, site-specific nutrient management, etc.^1,16–19^. In these strategies, the improvement of rice yield is mainly through increasing the number of fertilization, or regulating the use of panicle fertilizer at the stage of panicle development. However, different types of rice cultivars have different responses to N fertilizer application strategies^5,20^.

Phytohormones are a class of trace organic matter produced by plants themselves and have obvious physiological effects at very low concentrations^21,22^. At present, phytohormones include auxins (IAA), gibberellins (GAs), cytokinins (CTK), abscisic acid (ABA) and ethylene (ETH), and new phytohormones such as brassinosteroids (BRs), polyamines (PAs), jasmonates (JA), salicylic acid (SA) and strigolactone (SL)^23^. A large number of studies have shown that phytohormones play an important regulatory role in rice panicle development, grain filling, grain quality and quality, and different phytohormones play different roles and mechanisms. However, the biological mechanism of how phytohormones respond to N fertilization strategies to regulate rice N uptake and utilization efficiency, panicle morphogenesis and yield formation in different rice cultivars is still unclear. In the present study, a panicle weight type (PWT) rice cultivar, Dongfu 114 (DF114) and a panicle number type (PNT) rice cultivar, Longdao 11 (LD11) were used to compare the effects of N fertilization strategies on panicle development characteristics and yield of rice cultivars with contrasting yield types. This study will provide a theoretical basis for the rational and effective application of N fertilizer in rice production.

## Materials and methods

### Plant materials and growth conditions

The field experiments was conducted at the Acheng rice experiment base belonging to Northeast Agricultural University (45° 39′ N, 127° 34′ E) during the rice growing season in 2021, and repeated in 2022. The region is a cold temperate semi humid continental climate. The meteorological data such as temperature, rainfall, humidity and sunshine hours during the rice growing season in the experimental year were from the National Meteorological Data Center (Table 1). The soil texture of the test field is brown soil, and the soil nutrient content is shown in Table 2. Two rice cultivars with different yield types, DF114 and LD11 were used. The two rice cultivars have similar growth periods. The experimental materials were provided by the rice research group of Northeast Agricultural University.

**Table 1:** Monthly temperature (°C), rainfall (mm), relative humidity (%) and sunshine hours (h) during the growth seasonin 2021 and 2022.

Month	Average temperature (°C)				Rainfall (mm)		Average relative humidity (%)		Total sunshine	
	Minimum		Maximum						hour (h)	
	2021	2022	2021	2022	2021	2022	2021	2022	2021	2022
May	10.5	11	24.2	25.9	175.7	37.1	51.7	49.5	257.5	292.8
June	16.1	15.8	27.7	29.3	170.3	64.9	66.2	56.7	204	268.4
July	20.4	21.2	30	31.7	363.7	109.8	77.2	71.8	188.4	206.5
August	19.7	18.8	29.6	29.3	68.6	128.1	75.8	80.7	224.7	170.4
September	13.7	11.4	24.7	25.3	40.5	57.6	77.7	72.9	233.7	207.5

**Table 2:** Average values for selected soil characteristics of composite topsoil samples (0–20 cm) from the experimental fields in 2021 and 2022.

Year	Organic matter (g kg^−1^)	Total N (g kg^−1^)	Available P (mg kg^−1^)	Available K (mg kg^−1^)	pH
2021	17.3	1.09	32.13	109.11	6.63
2022	16.7	1.11	33.32	108.26	6.74

### Experimental design

The experiments were laid out in a complete randomized block design with three replicates. The N fertilizer strategies was the main plot treatment, and the rice cultivars formed the sub-plot treatment. Each plot was 8-m in length and 6-m in width with 30 cm row spacing and 13.3 cm terrace spacing, and two seedlings per hill. The main plots were separated by a berm. The seeds were sown on 16 April 2021, and 16 April 2022. At the four-leaf hpaniclet stage, seedlings with similar growth were selected and transplanted on May 18, 2021, and May 17, 2022. The experimental treatments included N0 (no N fertilizer), N1 (farmers routinely N fertilizer strategies, base fertilizer:tiller fertilizer of 6:4), N2 (base fertilizer : tiller fertilizer : panicle fertilizer of 6:2:2), N3 (base fertilizer:tiller fertilizer:panicle fertilizer of 5:3:2), N4 (base fertilizer:tiller fertilizer:panicle fertilizer of 4:3:3), a total of 5 treatments. The N was applied as urea(46%), at a rates were 150 kg ha^−1^. Phosphate fertilizer (P_2_O_5_) was applied once as a basal fertilizer at a rate of 90 kg ha^−1^. Potash fertilizer (K_2_O) was applied as a basal and panicle fertilizer at a ratio of 5:5, at a rate of 90 kg ha^−1^. With the exception of the different nitrogen fertilizer strategies, the other cultivation requirements were identical for all plots in both ypanicles. Chemicals were used to control weeds, diseases, and insects to prevent yield loss.

### Sampling and measurements

#### Nitrogen uptake and utilization

At the maturity stage, five representative hills were taken from each plot in 2021 and 2022. Aboveground plants sampled were separated into stems, leaves and grains. Dry weight of each part was determined by oven-drying at 80 °C to constant weight and weighed separately, and recorded the dry weight of each part. Tissue N content was measured with an elemental analyzer to calculate N uptake of each part (EA1110, Thermo Electon SPA., Italy). The calculation formula of N absorption and utilization related indicators is as follows:

$$ {\text{N accumulation }}\left( {{\text{NA}},{\text{ kg hm}}^{{ - {2}}} } \right) \, = {\text{ Aboveground dry matter weight at the mature stages }} \times {\text{ N concentration}}; $$

$$ {\text{N recovery efficiency }}\left( {{\text{NRE}}, \, \% } \right) \, = \, ({\text{NA}}\,{\text{of plant under}}\,{\text{N}}\,{\text{treatment }} - {\text{NA}}\,{\text{of plant under no}}\,{\text{N}}\,{\text{treatment}}) \, /{\text{N}}\,{\text{application rate }} \times { 1}00\% ; $$

$$ {\text{N physiological efficiency }}\left( {{\text{NPE}},{\text{ kg kg}}^{{ - {1}}} } \right) \, = \, ({\text{grain yield of plant under}}\,{\text{N}}\,{\text{treatment }} - {\text{ grain yield of plant under no}}\,{\text{N}}\,{\text{treatment}})/({\text{NA}}\,{\text{of plant under}}\,{\text{N}}\,{\text{treatment }} - {\text{NA}}\,{\text{of plant under no}}\,{\text{N}}\,{\text{treatment}}); $$

$$ {\text{N agronomic efficiency }}\left( {{\text{NAE}},{\text{ kg kg}}^{{ - {1}}} } \right) \, = \, ({\text{grain yield of plant under}}\,{\text{N}}\,{\text{treatment }} - {\text{ grain yield of plant under no}}\,{\text{N}}\,{\text{treatment}})/{\text{N application rate}} $$

$$ {\text{Partial factor productivity of applied}}\,{\text{N}}\left( {{\text{PFPN}},{\text{ kg kg}}^{{ - {1}}} } \right) \, = {\text{ grain yield of plant under}}\,{\text{N}}\,{\text{treatment}}/{\text{N}}\,{\text{application rate}}. $$

#### Grain yield and its components

In 2021 and 2022, grain yield (GY) and its components were measured at maturity. GY was measured from a harvest area of 1 m^2^ area for the three replicates in each plot, adjusted to 14% moisture. The yield components were measured for nine replicates in each plot. The panicle number from each samples were recorded to measure the effective panicles number (EP). The filled grains andunfilled grains per panicle were recorded to calculate the number of grains per panicle (GNP) and seed setting rate (SSR). The 1000-grain weight (TGW) was weighed and recorded.

#### Tillering dynamic survey

In 2021 and 2022, after 14 days of transplanting, 10 randomly selected plants were investigated every 7 days, and tillers were recorded until the number of tillers did not change. Calculate maximum tiller number (MTN) and productive tiller percentage (PTP) ccording to tillering dynamic survey.

#### Floret and grain development

At spikelet differentiation stage, pikelets differentiated number (SDN) were measured in 2021 and 2022. The differentiation of florets was observed using a planing microscope, and the SDN was recorded. The grain per panicle was used as the spikelets surviving number to calculate the spikelets degenerated number (SRN) and degraded percentage (RP).

$$ \begin{gathered} {\text{SRN }} = {\text{ SDN}} - {\text{spikelets surviving number}}; \hfill \\ {\text{RP }}\left( \% \right) \, = {\text{ SRN}}/{\text{SDN}} \times {1}00\% . \hfill \\ \end{gathered} $$

#### The panicle biomass and nitrogen concentration at panicle development stage

According to the heading stage data of the tested cultivars, this study sampled at five time points: 14 days before heading stage, 7 days before heading stage, heading stage, 7 days after heading stage and 14 days after heading stage, which were named S1, S2, S3, S4 and S5, respectively. The dry weight of each panicle was determined by oven-drying at 80 °C to constant weight and weighed separately, and recorded the panicle biomass. The panicle N concentration was measured with an elemental analyzer (EA1110, Thermo Electon SPA., Italy).

#### Panicle and root phytohormones content at panicle development stage

The panicles and roots per plot were sampled to determine phytohormones content were obtained at S1, S2, S3, S4 and S5 stages. The contents of IAA, ABA, GA and ZR were determined with enzyme-linked immunosorbent assay (ELIAS) method, according to the operation guide of ELIAS kit of China Agricultural University^24,25^.

### Statistical analysis

In this study, for the experimental variables, one-way analysis of variance (ANOVA) of SPSS 22.0 (SPSS Inc., Chicago, IL, USA ) was used to evaluate the differences between different N fertilizer application strategies. According to Fisher's LSD, there were significant differences between different N fertilizer application strategies at the p < 0.05 level. Graphs were drawn using edgeR software (http://www.r-project.org/) and Origin 2023b software (OriginLab, Northampton, MA, USA).

### Ethical statement

We ensure that all rice seeds used in this study originated from northeast agricultural university in Heilongjiang Province, China. The legality of these seeds complies with the IUCN Policy Statement on Research Involving Species at Risk of Extinction and the Convention on the Trade in Endangered Species of Wild Fauna and Flora. The rice seeds collected in the study are all cultivated rice in China rather than endangered and wild species. These varieties have passed the legal variety certification procedures in China and are licensed for production, planting, and market operations. The authors declare that the cultivation of plants and carrying out study in the Acheng rice experiment base of northeast agricultural university complies with all relevant institutional, national and international guidelines and treaties.

## Results

### Nitrogen uptake and utilization

As shown in Table 3, the indexes of N uptake and utilization in both cultivars were significantly different among different N treatments. Compared with farmers routinely N fertilizer strategies, the N fertilization strategies were significantly increased NRE, PFPN, NAE and NPE of two rice cultivars. The NRE, PFPN, NAE and NPE of DF114 were the highest under N4 treatment, and the NRE, PFPN, NAE and NPE of LD11 were the highest under N3 treatment. This result indicates that the N fertilization strategies could improve the N uptake and utilization of rice, and there were some differences among different yield types of rice cultivars.

**Table 3:** Effects of different N fertilization strategies on the nitrogen recovery efficiency (NRE, %), partial factor productivity of applied nitrogen (PFPN, kg kg − 1), nitrogen physiological efficiency (NPE, kg kg − 1), and nitrogen agronomic use efficiency (NAE, kg kg − 1) in rice cultivars in 2021 and 2022, with the results of the three-way ANOVA.

Year	Cultivars	Treatment	Nitrogen recovery efficiency (NRE, %)	Partial factor productivity of applied nitrogen (PFPN, kg kg^−1^)	Nitrogen agronomic use efficiency (NAE, kg kg^−1^)	Nitrogen physiological efficiency (NPE, kg kg^−1^)
2022	DF114	N0				
		N1	20.55d	42.33d	52.09d	39.37c
		N2	22.81c	44.59c	55.17c	41.35ab
		N3	23.72b	45.50b	57.67b	41.14b
		N4	24.90a	46.68a	58.77a	42.37a
	LD11	N0				
		N1	18.63c	41.65c	54.70c	34.06b
		N2	21.71ab	44.73b	56.54b	38.40a
		N3	22.58b	45.60a	58.47a	38.62a
		N4	21.09a	44.10b	56.58b	37.26a
2021	DF114	N0				
		N1	19.96b	42.76b	53.72d	36.97b
		N2	23.42a	46.22a	56.80c	41.02a
		N3	23.40a	46.20a	58.56b	39.77a
		N4	24.30a	47.09a	59.77a	40.47a
	LD11	N0				
		N1	20.29c	43.12c	52.93c	38.34c
		N2	23.28b	46.11b	54.40b	42.79b
		N3	24.59a	47.42a	56.01a	44.10a
		N4	23.31b	46.14b	55.43a	42.06b
F-value	Y		32.181**	103.838**	7.053*	46.558**
	C		43.129**	6.591*	69.647**	12.958**
	T		143.878**	204.269**	329.868**	57.94**
	Y * C		52.287**	13.296**	199.076**	173.043**
	Y * T		0.623 ns	0.873 ns	2.21 ns	1.266 ns
	C * T		12.726**	18.057**	59.435**	7.747**

The different lowercase letters represent significant among treatments at 0.05 level, respectively. * and ** means different at 0.05 and 0.01 level, respectively. N, nitrogen rate; C, cultivar; Y, year, the same as below.

### Grain yield and yield components

As shown in Table 4, the GY and yield components in two yield types of rice were significantly different among different N treatments. Compared with farmers routinely N fertilizer strategies, the N fertilization strategies were significantly increased GY and GPN of two rice cultivars. The GY and GPN of DF114 was the highest under N4 treatment, and the GY and GPN of LD11 was the highest under N3 treatment. Compared with farmers routinely N fertilizer strategies, the N fertilization strategies were significantly increased EP and SSR (except LD11 in 2022) of two rice varieties. The EP of two rice cultivars were the highest under N3 treatment. Compared with farmers routinely N fertilizer strategies, the N fertilization strategies were decreased TGW of two rice cultivars (except LD11 in 2022). Interestingly, increase the application ratio of panicle N fertilizer can guarantee the GPN and SSR of rice, but excessive N fertilizer postponing reduces the EP of PNT rice cultivar LD11.

**Table 4:** Effects of different N fertilization strategies on the yield components of rice cultivars in 2021 and 2022, with the results of the three-way ANOVA.

Year	Cultivars	Treatment	Grain yield (GY, t ha)	Effective panicles (EP, per m^−2^)	Grain per panicle (GPN)	Seed setting rate (SSR, %)	1000-grain weight (TGW, g)
2022	DF114	N0	3.92e	150.95e	125.29e	90.32a	23.42ab
		N1	7.62d	279.87d	126.63d	85.82bc	23.91a
		N2	8.03c	310.41c	133.68c	85.31c	22.9bc
		N3	8.19b	340.87a	139.52b	86.92b	23.08b
		N4	8.4a	320.9b	143.07a	89.17a	22.38c
	LD11	N0	4.14d	179.77d	120.42a	90.28a	21.99b
		N1	7.5c	360.23c	111.06d	86.56b	22.88a
		N2	8.05b	394.33b	114.61c	86.92b	22.66a
		N3	8.21a	421.08a	117.7b	87.53b	22.74a
		N4	7.94b	417.00a	114.25c	87.92b	22.54a
2021	DF114	N0	4.1d	149.28e	132.48c	90.54a	23bc
		N1	7.7c	308.87d	131.5c	83.48b	23.62a
		N2	8.25b	345.6b	138.43b	84.86b	23.48a
		N3	8.28b	366.08a	139.49b	89.45a	22.75c
		N4	8.48a	334.02c	143.75a	90.72a	23.33ab
	LD11	N0	4.11d	190.72d	113.28b	91.23a	21.91c
		N1	7.76c	343.3c	109.7c	83.4c	22.52a
		N2	8.3b	383.74b	112.61b	84.64c	22.3ab
		N3	8.54a	397.74a	117.83a	84.66c	22.31ab
		N4	8.31b	387.37b	117.63a	87.46b	22.01bc
F-value	Y		113.91**	21.74**	11.84**	7.97**	4.99*
	C		1.53 ns	4261.99**	4511.56**	7.2*	195.18**
	T		8139.46**	7707.18**	145.05**	91.62**	17.83**
	Y * C		5.48*	414.66**	64.25**	17.48**	15.17**
	Y * T		3.43*	8.62**	1.95 ns	8.11**	4.41**
	C * T		19.4**	64.25**	69.4**	8.38**	7.65**
	Y * C * T		6.67**	47.71**	23.87**	5.1**	8.2**

### Dynamic changes of tillers

**Figure 1:**
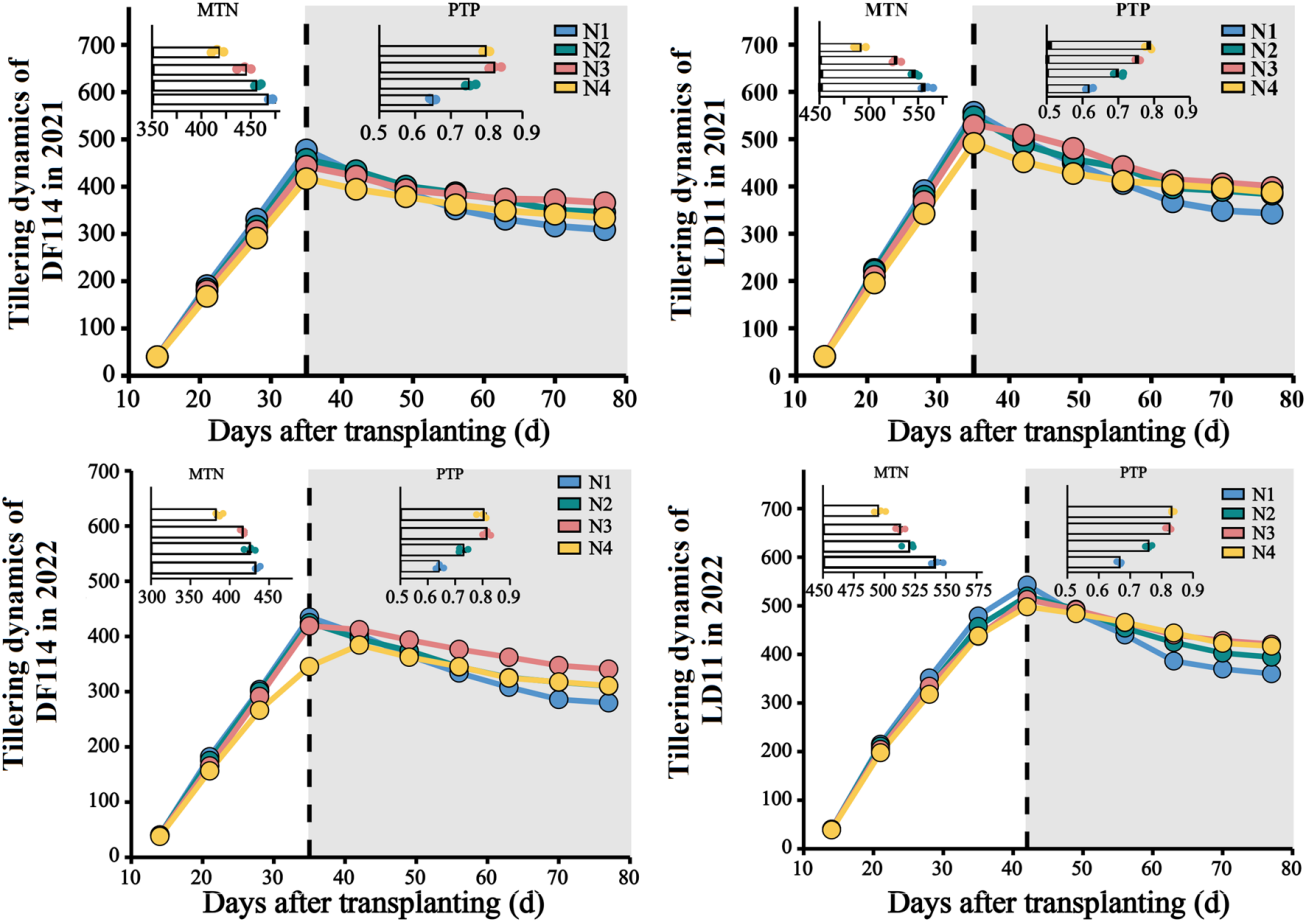
Tillering dynamics of rice under different treatments in 2021 and 2022. *MTN* maximum tiller number, *PTP* productive tiller percentage.

As shown in Fig. 1, the N fertilization strategies significantly affected the tillering of rice, and the tillering dynamic trends of DF114 and LD11 were basically the same under among N fertilization strategies. At 20–30 days after transplanting, the number of tillers increased rapidly and reached the highest number of tillers at about 35 days (LD11 reached the highest number of tillers at about 42 days in 2022). After 50 days of transplanting, the growth center of the plant was transferred to the stem and panicle, and the nutrients transported to the tillers were greatly reduced. The new tillers did not occur, and some of the small tillers that had been born began to die in succession until the number of tillers in the population tended to be stable after heading. In this study, compared with the farmers routinely N fertilizer strategies, the N fertilization strategies were significantly reduced the MTN of rice, but also significantly increased the PTP of rice to ensure a higher EP of rice.

### Floret and grain development

The spikelets differentiation and degeneration, and grain development in two yield types of rice were significantly different among different N treatments (Table 5). Compared with the farmers routinely N fertilizer strategies, the N fertilization strategies were significantly increased SDN of two rice cultivars (except DF114 in 2022). Compared with the farmers routinely N fertilizer strategies, the N fertilization strategies were significantly decreased SRN and RP of two rice cultivars. The SRN and RP of DF114 were the lowest under N4 treatment, and the GPN of LD11 was the lowest under N3 treatment.

**Table 5:** Effects of different N fertilization strategies on the floret growth and development of rice in 2021 and 2022, with the results of the three-way ANOVA.

Year	Cultivars	Treatment	Spikelets differentiated number (SDN)	Spikelets degraded number (SRN)	Spikelets surviving number	Degraded percentage (RP)
2022	DF114	N0	187.27a	61.98a	125.29d	0.33a
		N1	183.01a	56.38b	126.63d	0.31b
		N2	186.49a	52.81c	133.68c	0.28c
		N3	186.46a	46.94d	139.52b	0.25d
		N4	184.42a	41.35e	143.07a	0.22e
	LD11	N0	167.42a	47a	120.42a	0.28a
		N1	154.63c	43.57b	111.06d	0.28a
		N2	155.29c	40.68c	114.61c	0.26bc
		N3	157.82b	40.12c	117.7b	0.25c
		N4	154.95c	40.71c	114.25c	0.26b
2021	DF114	N0	183.21b	50.73b	132.48c	0.28b
		N1	185.81ab	54.31a	131.5c	0.29a
		N2	187.15a	48.72b	138.43b	0.26c
		N3	188.08a	48.59b	139.49b	0.26c
		N4	186.06a	42.3c	143.75a	0.23d
	LD11	N0	155.56bc	42.28b	113.28b	0.27c
		N1	154.28c	44.58a	109.7c	0.29a
		N2	157.71b	45.11a	112.61b	0.29ab
		N3	157.73b	39.89c	117.83a	0.25d
		N4	163.47a	45.84a	117.63a	0.28bc
F-value	Y		0.07 ns	8.29**	11.84**	5.56*
	C		3986.71**	542.63**	4511.56**	0.22 ns
	T		8.96**	95.54**	145.05**	171.19**
	Y * C		0.83 ns	40.88**	64.25**	72**
	Y * T		23.94**	34.19**	1.95 ns	25.14**
	C * T		8.52**	55.11**	69.4**	77.58**
	Y * C * T		7.73**	7.63**	23.87**	14.64**

### The relationships among grain yield and the floret and grain development

**Figure 2:**
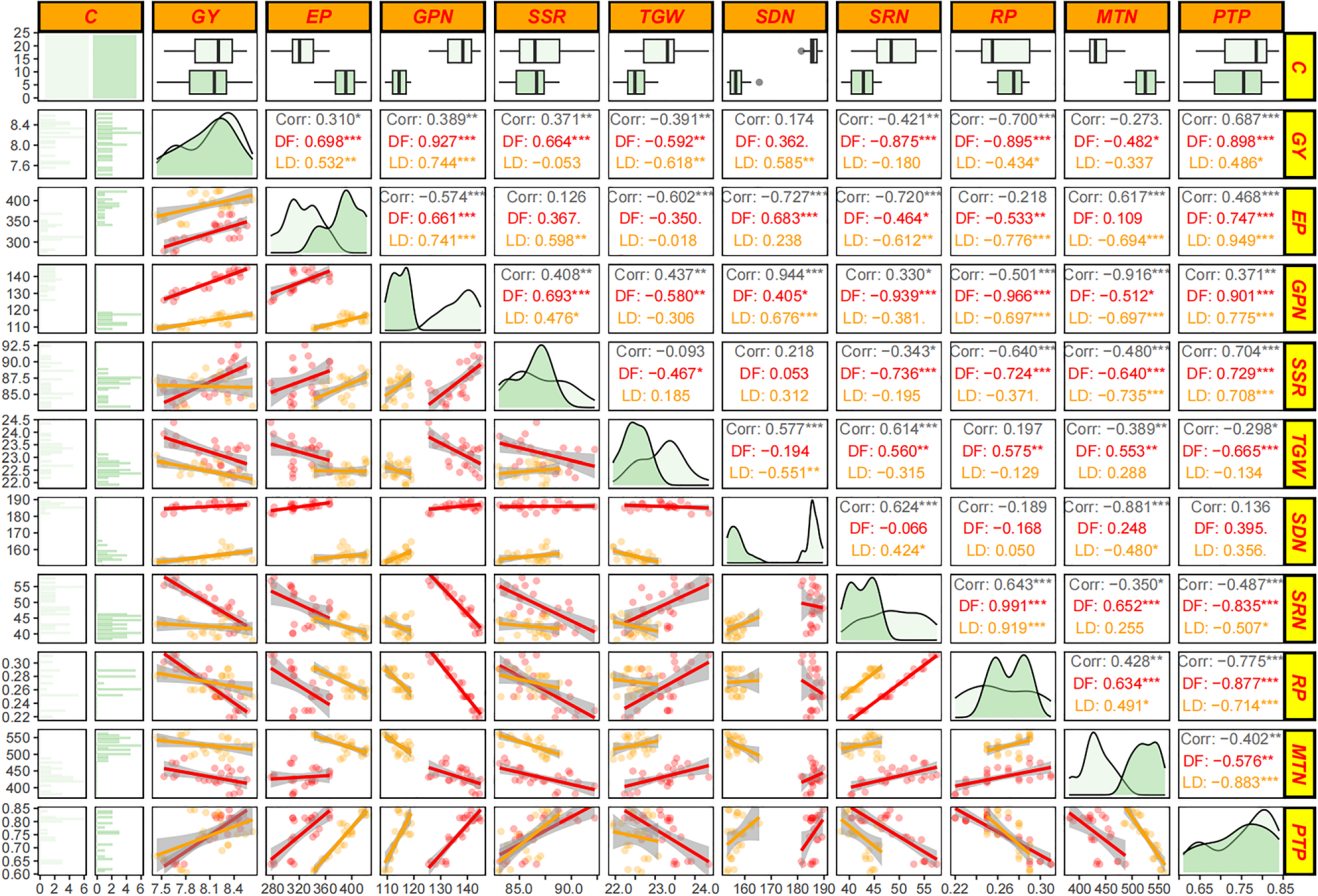
The relationships among grain yield and the floret and grain development. The *, ** and *** indicate that at the level of 0.05, 0.01 and 0.001, respectively. *GN* grain yield, *EP* effective panicles, *GPN* grain per panicle, *SSR* seed setting rate, *TGW* 1000-grain weight, *SDN* spikelets differentiated number, *SRN* spikelets degraded number, *RP* degraded percentage, *MTN* maximum tiller number, *PTP* productive tiller percentage.

The formation of rice GY mainly depends on the number of EP, GPN, SSR and TGW. As shown in Fig. 2, the EP and GPN were significant positively, and TGW was significant negatively correlated with GY in both rice cultivars. The SSR were significant positively with GY of DF114. The farmers routinely N fertilizer strategies can produce more tillers, but there are more ineffective tillers. Correlation analysis showed that PTP was an important basis for higher EP to ensure higher yield. The differentiation and degeneration of spikelets determine the GPN of rice. Correlation analysis showed that the SRN was significant negatively correlated with GPN of DF114, and the SDN was significant positively correlated with GPN of LD11. These results indicated that panicle and grain development was the main factor responsible for the difference in yield among two rice cultivars under difference N treatments.

### The panicle biomass and nitrogen concentration at panicle development stage

**Figure 3:**
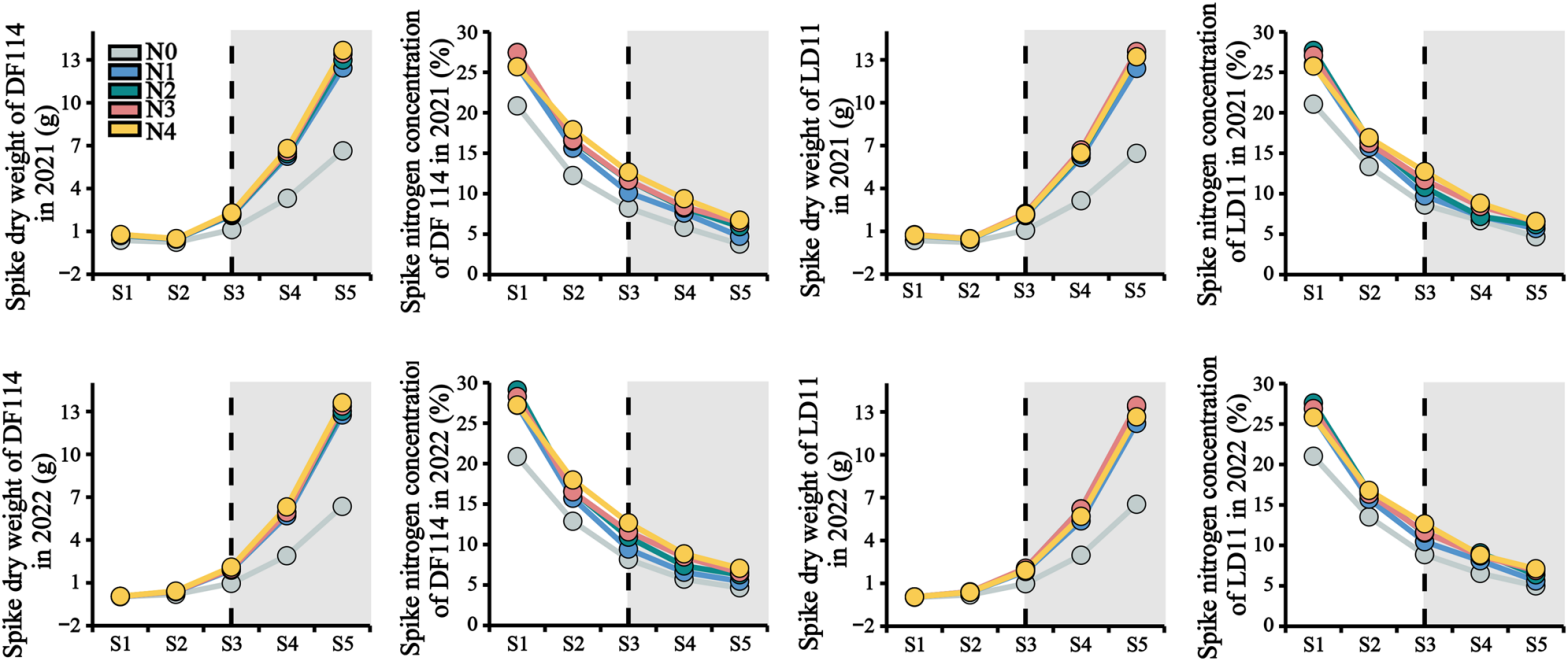
The N concentration and panicle dry weight of the rice under different treatment during critical period.

At the panicle development stage, the responses of different yield type rice cultivars to N fertilizer treatments were coincident (Fig. 3). With the development of panicle, the N concentration of young panicle decreased and the biomass increased. Compared with the farmers routinely N fertilizer strategies, the N fertilization strategies were significantly increased panicle biomass (PB) of two rice varieties during the period from S3 to S5, the panicle biomass of DF114 was the highest under N4 treatment, and the panicle biomass of LD11 was the highest under N3 treatment. Compared with the farmers routinely N fertilizer strategies, the N fertilization strategies were significantly impacted panicle N concentration (PN) of two rice varieties during the period from S1 to S5.

### The phytohormone content in panicle and root at panicle development stage

**Figure 4:**
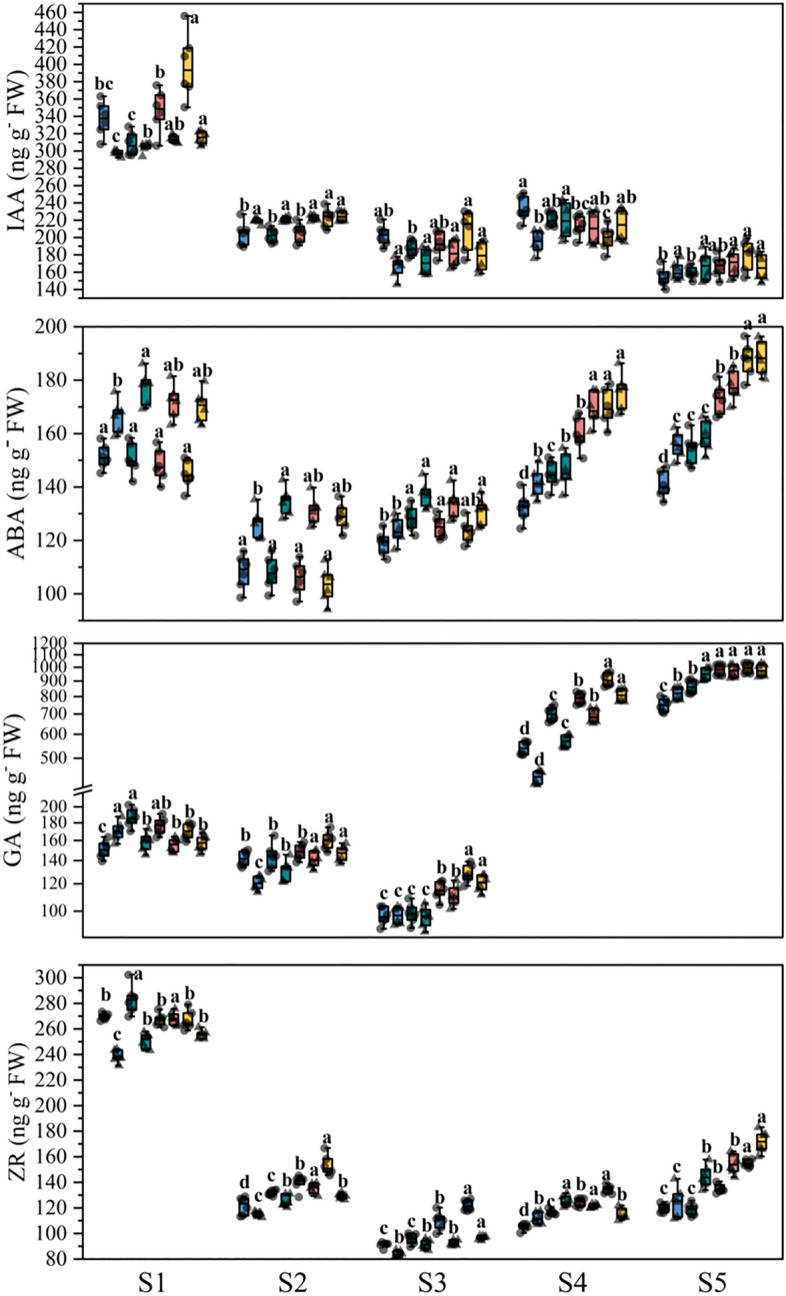
The phytohormones content in panicle at panicle development stage. Result is the average from 2021 to 2022. The different small letters above the box indicate significant difference in the same panicle differentiation stage among the same cultivars at P < 0.05. From left to right represent N1, N2, N3 and N4, respectively.

The IAA content in the panicle of tow rice cultivars gradually decreased with the developmental stage of the young panicle under different N treatments. The ABA content in the panicle of tow rice cultivars were decreased first and then increased with the developmental stage of the young panicle under different N treatments. Compared with the farmers routinely N fertilizer strategies, the N fertilization strategies were significantly increased the ABA content of DF114 panicle at S3-S5 stages, and increased the ABA content of LD11 panicle at S1-S5 stages. The GA content in the panicle of tow rice cultivars were decreased first and then increased with the developmental stage of the young panicle under different N treatments. Compared with the farmers routinely N fertilizer strategies, the N fertilization strategies were significantly increased the GA content of two rice cultivars panicle at S3–S5 stages. The ZR content in the panicle of tow rice cultivars were decreased first and then increased with the developmental stage of the young panicle under different N treatments. Compared with the farmers routinely N fertilizer strategies, the N fertilization strategies were significantly increased the ZR content of DF114 panicle at S2-S5 stages, and increased the ZR content of LD11 panicle at S1, S2 and S5 (Fig. 4).

**Figure 5:**
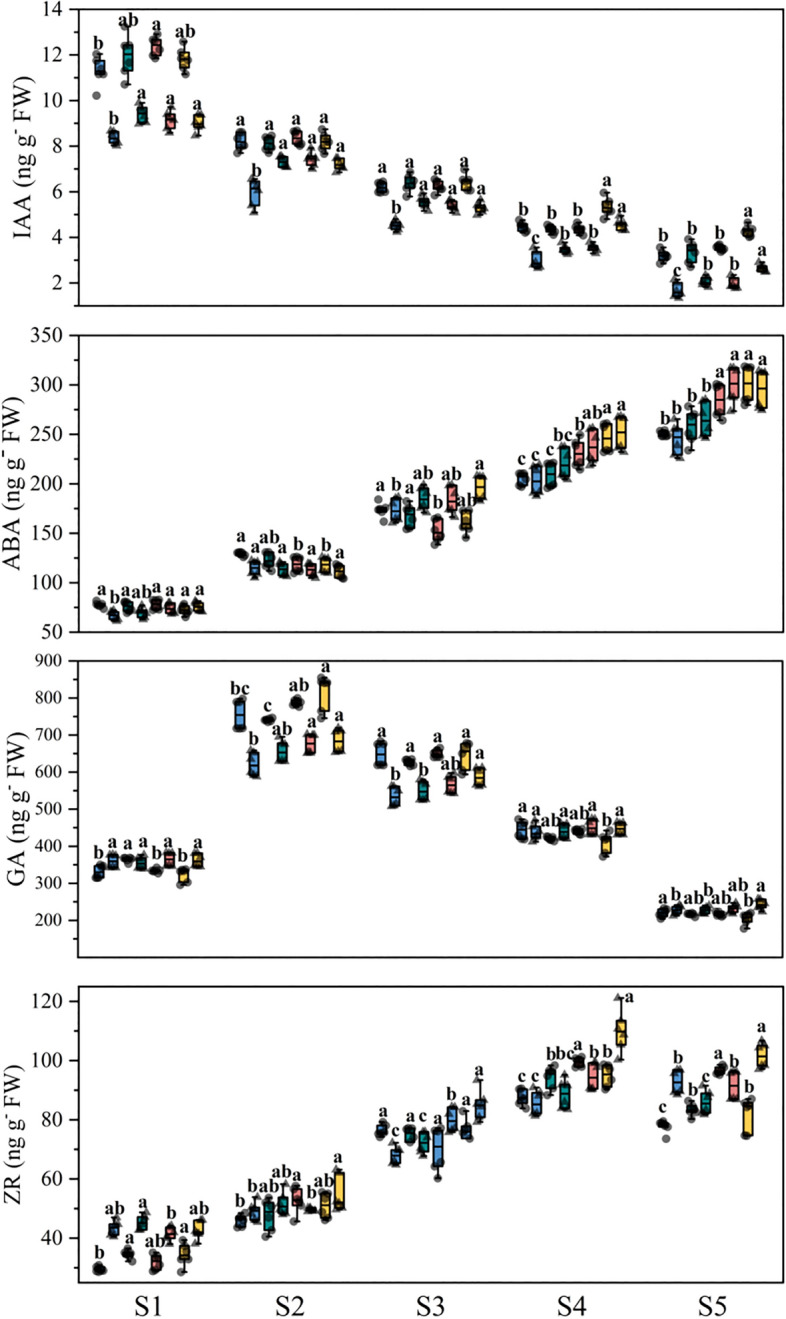
The phytohormone content in root at panicle development stage. Result is the average from 2021 to 2022. The different small letters above the box indicate significant difference in the same panicle differentiation stage among the same cultivars at P < 0.05. From left to right represent N1, N2, N3 and N4, respectively.

The IAA content in the root of tow rice cultivars gradually decreased with the developmental stage of the young panicle under different N treatments. Compared with the farmers routinely N fertilizer strategies, the N fertilization strategies were significantly increased the IAA content of DF114 root at S1 stages, and increased the IAA content of LD11 root at S1–S5 stages. The ABA content in the root of tow rice cultivars were increased with the developmental stage of the young panicle under different N treatments. Compared with the farmers routinely N fertilizer strategies, the N fertilization strategies were significantly increased the ABA content of DF114 root at S4 and S5 stages, and increased the IAA content of LD11 root at S3-S5 stages. The GA content in the root of tow rice cultivars were increased first and then decreased with the developmental stage of the young panicle under different N treatments. The ZR content in the root of tow rice cultivars were increased with the developmental stage of the young panicle under different N treatments. Compared with the farmers routinely N fertilizer strategies, the N fertilization strategies were significantly increased the ZR content of DF114 root at S2 and S5 stages (Fig. 5).

### Correlations between panicle and grain development, and panicle and root phytohormones

**Figure 6:**
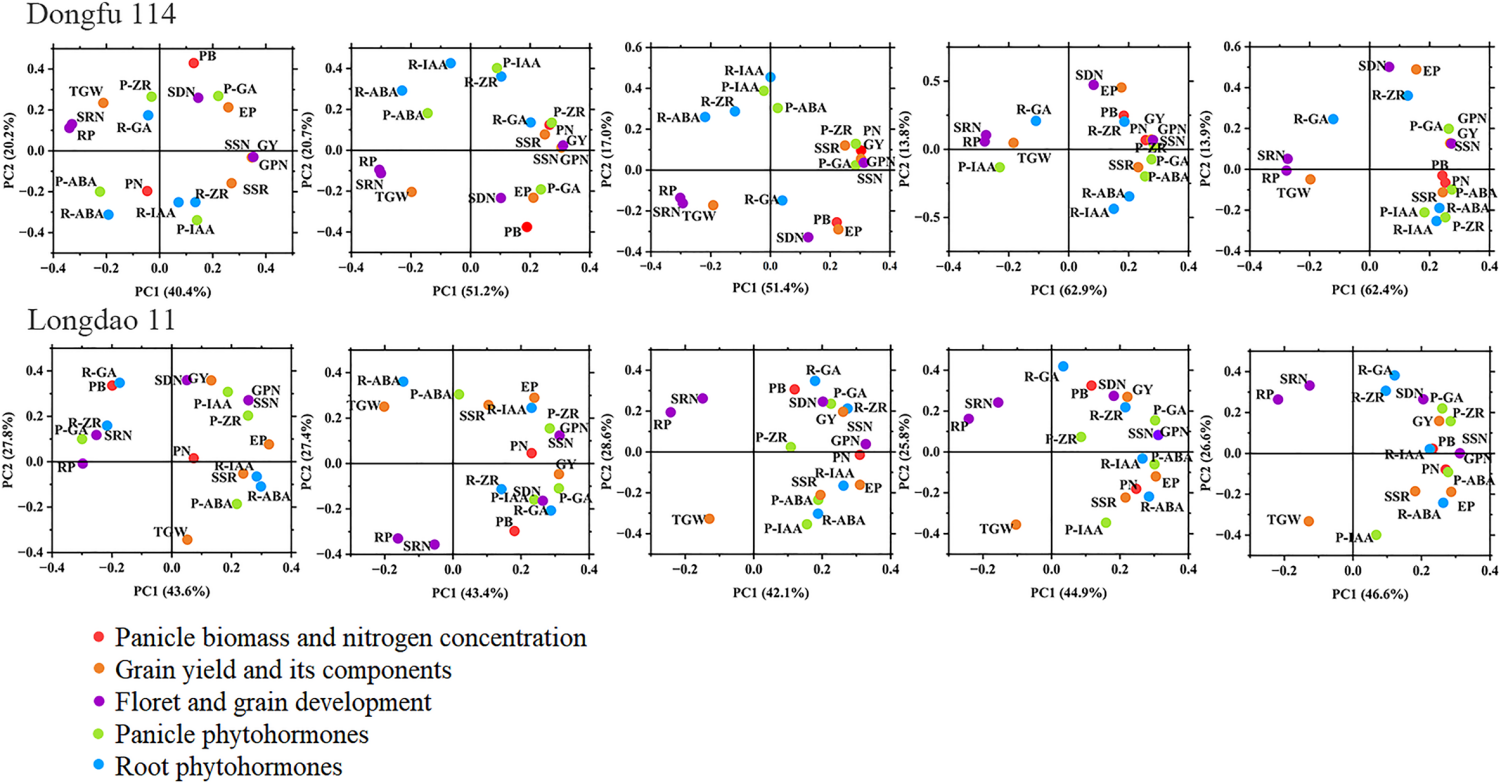
Principal component analysis (PCA) of panicle differentiation and development traits, panicle and root phytohormones determined on rice under different treatment during critical panicle differentiation stages.

The results of correlations analysis (Figs. 2, 6) showed that the spikelet differentiation and grain filling were the main reasons for the different responses of the two rice cultivars to N treatments. The results of principal component analysis show that the SRN of DF114 were significantly negatively correlated with P-ZR, P-GA and PN at panicle differentiation stage. The SSR of DF114 were significantly positively correlated with P-ZR, P-ABA and R-ABA at after flowering stage. The SDN of LD11 were significantly positively correlated with P-IAA at panicle differentiation stage. These results indicate that phytohormones play an important role in rice yield formation, and reasonable optimization of N fertilizer application strategies can regulate rice phytohormone levels to improve NUE and yield.

## Discussion

The N is one of the most important nutrient elements in the process of rice growth, and the amount of N fertilizer significantly affects the yield of rice^17^. Farmers usually apply most of the N fertilizer at the early stage of nutrition. At this time, the absorption capacity of rice roots to N is limited, and a large amount of N is retained in soil and irrigation water. The results of ^15^N tracing shown that the NUE of N fertilizer applied to rice before regreening was lower, and a large amount of N fertilizer was lost and volatilized^26,27^. In this study area, farmers applied 150 kg hm^−2^ of N fertilizer. Although the amount of fertilizer applied is less than some areas in southern China, there is still a large amount of the N fertilizer applied at the initial stage of tillering. Some studies have shown that an appropriate increase in the proportion of panicle fertilizer can improve the NUE and yield of rice^28,29^. In this study, higher panicle fertilizer ratio can effectively improve the NUE and yield of both rice cultivars. However, there were some differences among different cultivars, DF114 had the highest NUE and yield under N4 treatment, and LD11 had the highest NUE and yield under N3 treatment. This result was in agreement with previous observations that rice cultivars with different productivity and NUE can achieve maximum yield under precise quantitative cultivation^19,30,31^. It can be seen that although the optimized N fertilizer application strategies can improve the NUE of rice, the response of rice varieties with different yield types to the optimized N fertilizer application strategies is different. The increase of panicle N fertilizer proportion is more conducive to improving the NUE and yield of PWT rice cultivars.

The effects of N application strategies on rice NUE and yield were related to the response characteristics of multiple yield components to N application strategies^32^. Tillers are composed of effective tillers and ineffective tillers. Excessive ineffective tillers not only consume nutrients, but also deteriorate population permeability and aggravate the occurrence of pests and diseases^33^. Previous studies have found that the PTP of rice is closely related to a number of population quality indicators, and it is an intuitive and comprehensive indicator for diagnosing population quality^17–19^. Under normal circumstances, rice plants have a stable law of leaf and tiller extension. However, excessive N application in the early stage of rice growth will shorten the time interval of leaf emergence, accelerate the occurrence of tillers, and increase the number of ineffective tillers. On the contrary, insufficient N fertilizer will also lead to a longer time interval for leaf emergence, a slower rate of tillering, and insufficient panicles^34^. In this study, postponing N fertilizer significantly reduced the MTN of rice and significantly increased the spike rate. However, the further increase of panicle fertilizer ratio also significantly reduced the number of effective panicles of LD11. Therefore, under the premise of ensuring the number of panicles, it is an important way to improve rice NUE and yield an by reducing the seedling peak, reducing the ineffective tillers and increasing the PTP.

The reproductive development process of crops is closely related to the formation of yield. Many studies have suggested that the key to further increase the yield of major grain crops such as rice is to increase the GPN^35^. The development stage of young panicle is the key period to determine the number of spikelets per panicle of rice, including rachis differentiation stage, primary and secondary branch differentiation stage and spikelet differentiation stage^36^. The number of spikelets is the basis of the number of filled grains, which together with the SSR determines the GPN of rice^37^. According to previous studies, the application of N fertilizer at the panicle differentiation stage of rice can promote floret differentiation^38,39^. In this study, the response of floret differentiation of the two varieties to N fertilizer management patterns was quite different. The SDN of PWT varieties was significantly increased with the increase of panicle N fertilizer ratio. Compared with farmers routinely N fertilizer strategies,, the increase of panicle fertilizer ratio significantly reduced the SRN and RP, and then increased the GPN and expanded the sink capacity, which was conducive to improving N absorption and utilization efficiency and yield.

In order to illustrate the biological basis of the response of panicle traits to N fertilization strategies, this study measured the panicle N and phytohormines content at the main stage of panicle development. Phytohormines play a essential role in regulating the development of plant organs, nutrient absorption and transport, and defensive adaptation to stress^40,41^. IAA is the most bizarre phytohormines discovered so far. Its content is very low in plants, but it plays an important role in crop organogenesis and morphogenesis, tissue differentiation tendency and apical dominance^42–44^. High N concentration in differentiated organs is conducive to promoting biosynthesis of IAA and differentiation of tissues and organs^45^. ABA is called senescence hormone, which can induce the occurrence of plant senescence, but the role of ABA in the senescence process is contradictory. Some studies have shown that ABA can coordinate the senescence process and ensure food production in the stage of crop yield formation^46–48^. ZR is a plant endogenous hormone belonging to cytokinin. It can accelerate cell division and plays an important role in regulating the size and activity of meristem, and directly affects the initiation and development of reproductive organs^49^. The rice LONELY GUY (LOG) gene encodes a cytokinin-activating enzyme that regulates the biosynthesis of cytokinin in rice. It is an important regulatory factor necessary for maintaining the activity of meristem. The meristem development of the log mutant is terminated in advance, the number of branches and spikelets is significantly reduced, and the floral organ cannot start normally^50^. Correlation analysis showed that SDN and SSR were the main factors affecting yield of DF114. PCA results showed that ZR content in panicle of DF114 was significantly correlated with N concentration in panicle, and was significantly negatively correlated with SRN and RP in floret differentiation stage. ABA content in panicle was significantly positively correlated with SSR in grain filling stage. The floret differentiation of LD11 was the main factor affecting the yield. PCA results showed that IAA in the panicle of LD11 was significantly positively correlated with SDN in the floret differentiation stage. These results suggest that N fertilizer strategies can affect the content of phytohormones in rice at the panicle differentiation stage, and then regulate the differentiation and development of rice panicles to affect yield. It is of great significance to optimize the application mode of N fertilizer according to the characteristics of varieties to improve rice yield and ensure food security.

## Conclusions

Compared with farmer fertilization, N fertilizer application strategy significantly improved the NUE and yield of rice, but the response of rice varieties with different yield types to N fertilizer application strategy was different. Correlation analysis showed that panicle development related indexes SDN, SRN, RP and SSR were the main factors affecting NUE and yield of PNT and PWT rice varieties.The results showed that in the early stage of panicle development, the higher IAA content in the panicle of PNT rice varieties was beneficial to promote the differentiation of spikelets to ensure the yield.. While the indexes of SRN, RP and SSR in the middle and late stages of panicle development of PWT rice varieties were significantly correlated with yield. These results will lay a theoretical foundation for guiding the construction of high-yield and high-efficiency cultivation models for different yield types of rice varieties.

## Data Availability

The datasets used and/or analysed during the current study available from the corresponding author on reasonable request (Q.Z., qiangz@jlau.edu.cn).
